# A new technique for precisely and accurately measuring lumbar spine bone mineral density in mice using clinical dual energy X-ray absorptiometry (DXA)

**DOI:** 10.1080/15376510802499030

**Published:** 2009-06-30

**Authors:** Ranjitha Katikaneni, Adharsh Ponnapakkam, Eric Miller, Tulasi Ponnapakkam, Robert C. Gensure

**Affiliations:** Endocrinology Research Laboratory, Ochsner Clinic Foundation, New Orleans, LA, USA

**Keywords:** Accuracy, Bone mineral density (BMD), DXA, Mice, Precision

## Abstract

Dual Energy X-ray Absorptiometry (DXA) is effective in measuring bone mineral density (BMD) in mice for early detection of osteoporosis. However, scanners designed for use with small animals (i.e. PIXImus) are very expensive. Used human DXA machines are cheaper to obtain, but analysis of scans from these instruments is operator-dependent. Obtaining reliable data depends on having a single operator analyze the scans in a blinded fashion. Scan quality is improved by excising the bone prior to scanning, which does not allow serial measurements. This study describes a novel method of analyzing lumbar spine BMD in mice using whole body DXA. This non-invasive technique has a high degree of precision and reproducibility, with good correlation between multiple observers. Inter-observer variability (0.063 ± 0.00317 g/cm^2^ [mean ± SD], 5.05 [% coefficient of variation (CV)], repeat scan variability (0.063 ± 0.00364 g/cm^2^ [mean ± SD], 5.94 [%CV]) were very low compared to variability between different animals (0.063 ± 0.00588 g/cm^2^ [mean ± SD], 9.64 [%CV]) and variability seen in same animal over time (0.011 ± 0.00885 g/cm^2^ [mean ± SD], 80.68 [%CV]). The measurement error is thus smaller than the biological variation. Accuracy was determined by comparing average peak BMD from two scans per mouse in-vivo (0.066 g/cm^2^) versus excised spine (0.065 g/cm^2^). Furthermore, correlation between bone ash weights and whole body lumbar spine BMD measurements (*p* < 0.0001) was highly significant. This technique thus shows a high degree of precision and accuracy, even with multiple observers, for measuring BMD in mice using a DXA machine designed for clinical use.

## Introduction

The measurement of bone mass is an essential step in assessing bone loss in both humans and animals. Several non-invasive techniques are available for analyzing bone mineral density and bone mineral content. These measurements provide the essential information for identifying high risk groups for osteoporosis and for assessing the fracture-risk of individuals (Cheng et al. 1997; Gala Paniagua et al. 1998; Di Leo et al. 2002). Dual Energy X-Ray Absorptiometry (DXA) is currently the most widely used non-invasive technique to assess bone mineral density and bone mineral content in human and animal research. DXA was first brought into clinical practice in the 1980s and is now a common tool to assess bone loss in humans (Lochmuller et al. 2001). It has also recently been adapted to measure body composition in several types of animals, such as rats, by using specialized software in conjunction with DXA machines designed for clinical use (Nagy and Clair 2000).

Mice are an especially useful model in research because of their relatively high genetic homogeneity (Iida-Klein et al. 2003). The mouse carries virtually all genes that operate in humans, enabling us to develop mouse models for a variety of human disorders (Hofker 2003). Genetically modified mice are an excellent tool in present day biomedical research, as they provide an excellent approach for unraveling gene function at the cellular level (Vonken and Hofker 2005). In addition, mice are inexpensive, small, and easy to maintain in the laboratory. They also have a short breeding cycle with a known genetic background (Moore 1995).

Old clinical DXA machines are cheaper to obtain for research purposes. Old instruments (DXA) are frequently replaced in clinical practice, and can be purchased for use in research at greatly reduced prices. Similar DXA instruments with the same resolution produce comparable results. However, these machines are optimized for human use. The image quality is generally poorer when used with mice because of the smaller size and areal density of the spine, and the provided software does not automatically produce a BMD measurement which is accurate or reliable. Some machines can be modified for use with small animals (special X-ray emitters/detectors and/or special software), but this adds to the cost. Even with these modifications, the analysis of the BMD has to be performed manually, which can lead to significant inter-observer variation and introduce bias into the dataset. Most studies using these subjective analysis techniques employ a single blinded observer to avoid bias in the BMD analysis; however, this limits the number of animals which can be used in such studies.

A specialized DXA machine called Lunar PIXImus (General Electric, Madison, WI) has been developed specifically for measuring BMD in small animals, such as mice. The total body short-term precision in-vivo and in-vitro for this machine is very high, according to the manufacturer's specification. The total body short-term precision in-vivo, in terms of percentage coefficient of variation (% coefficient of variation (CV)), is 0.87% and ex-vivo is 0.5% (Iida-Klein et al. 2003). While it is specifically designed for BMD measurements in small animals, this equipment is very expensive. The analysis of BMD is highly dependent upon image quality, as the software attempts to map the bone–soft tissue interface, similar to the techniques used to analyze DEXA scans in humans. There is again significant inter-observer variation in the analyses, and there is potential to introduce bias into the dataset.

This study examines the precision and accuracy of a novel technique for measuring bone mineral density of the lumbar spine in mice, in-vivo, using an unmodified clinical DXA machine (Hologic QDR-1000 plus). Rather than relying on subjective or computational assessment of the location and boundaries of the lumbar spine in obtaining our DEXA measurement, we instead performed a ‘brute force’ analysis of one-pixel wide strips which are parallel to the spine and are placed across a region of interest which includes the lumbar spine. We then record the highest BMD measurement obtained as the BMD of the animal. We have found that this measure is reliable and correlates well with BMD measurements obtained using the traditional method of analysis on the excised spine from the same animal, as detailed below.

Unlike previous techniques, this new technique is objective and is less dependent upon image quality. It can thus be used with any scanner which has sufficiently high resolution (at least 0.095 cm pixels), with or without modifications for use with small animals. The scans are performed on whole animals, rather than on excised bones. There is therefore no need to sacrifice the animals to obtain the measurement, and time course studies can be more easily performed. There is no possibility of introducing bias into the BMD measurements, so the analysis need not be performed in a blinded fashion. The measured BMDs from this technique correlate well with bone ash weights and with bone mineral density measurements from excised spines. Furthermore, we found that there was high correlation between BMD measurements obtained from the same scans by multiple observers. Larger studies can thus be performed, with DEXA analyses by multiple observers pooled together.

## Materials and methods

### Animals

Female C57BL/6J mice 3–5 weeks old were obtained from Jackson Laboratory (Bar Harbor, ME). Institutional animal care approval was obtained from the Ochsner Clinic Foundation to carry out the studies with mice. The mice were then acclimated for 2 weeks in the animal room. They were exposed to a 12/12 hour light/dark period at a temperature of 20–21°C. The mice were given access to tap water and were given a diet consisting of 18% protein purchased from Harlan Company, located in Barton (IL) and Madison (WI). A total of 20 mice were used in the study: two separate sets of animals were obtained 3 months apart (eight mice in Set 1 and 12 mice in Set 2). Where the same measurements were obtained at the same age in each set, the results are pooled between the two groups.

### X-ray bone densitometer

**Figure 1:**
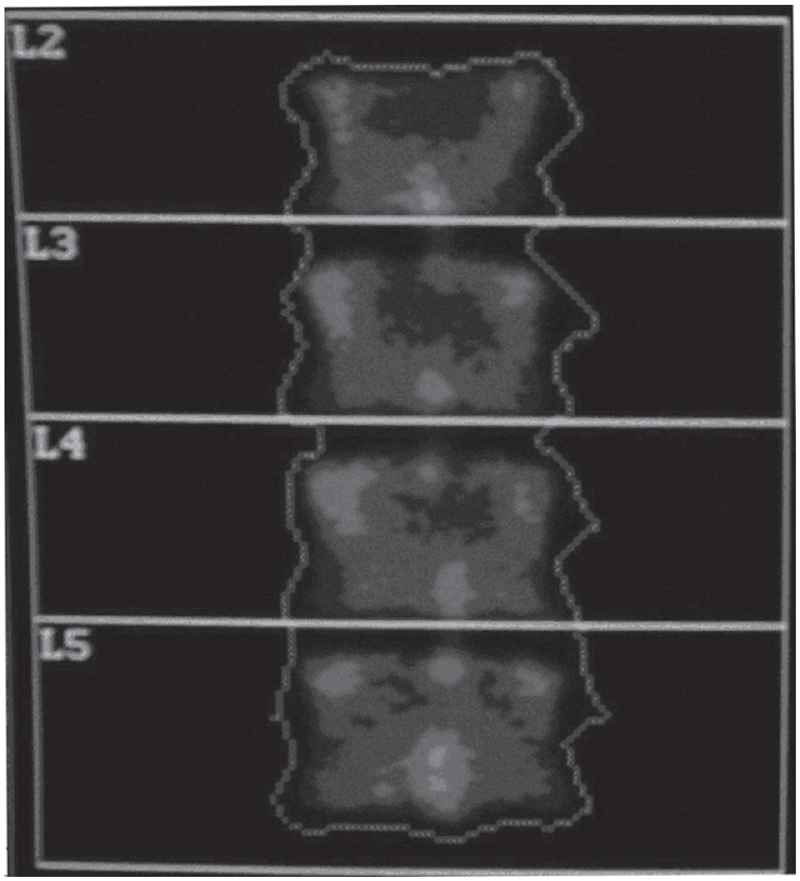
Bone mineral image with standard region of interest including L2–L5, is done at the start and end of the experiment as an independent check of system calibration.

**Figure 2:**
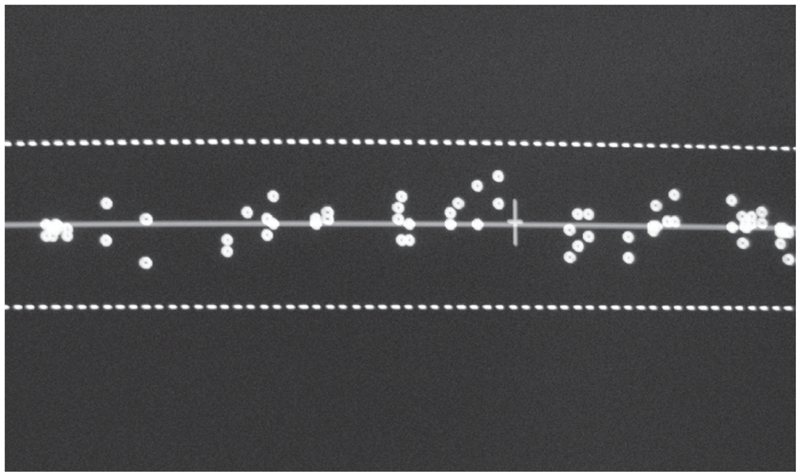
Quality control graph from a QDR 1000 plus, showing an accurate performance of the machine over an extended period of time.

A Hologic QDR-1000 plus machine, which was salvaged from a nearby hospital immediately after hurricane Katrina, was relocated by the manufacturer to our laboratory. The machine was calibrated and validated for its performance by the local representative of the company. The spine phantom was scanned according to the manufacturer's guideline at the start and end of the scanning session to verify proper system calibration (Figure 1). For quality control, the values were plotted and compared with the QC data base that was supplied by the manufacturer, which appears as a red line on the plot. Dashed limit lines indicate a + or −1.5% range of variation about the mean. BMD values outside these limits indicate a problem with the system. Daily measurements of spine phantom BMD are added to this plot. Our results were consistently within the dashed lines, indicating accurate performance of the machine (Fig. 2).

Scans from mice were obtained using the standard hardware configuration and the installed software (which is optimized for human use); the only modification was removal of the cushion pad prior to obtaining scans. The machine was used in a high-resolution mode with the following parameters for the scans:

1. Length of scan = 4 cm;
2. Width of scan = 4 cm;
3. Line spacing = 0.100 cm; and
4. Point resolution = 0.095 cm.

**Figure 3:**
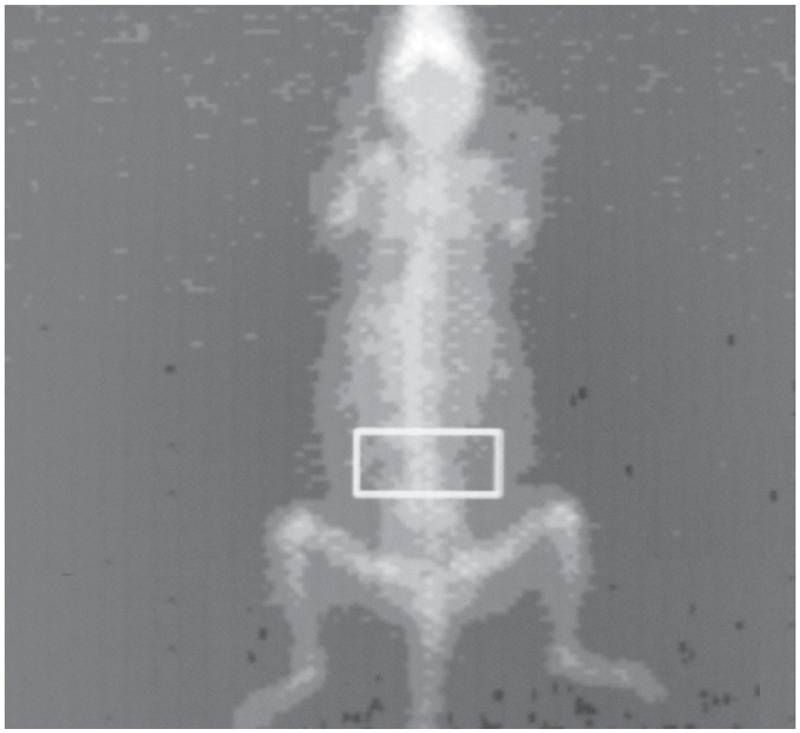
Positioning of the mice. Mouse is placed in prone position perpendicular to the axis of scanning, with the hind legs extending away from the body showing the standard box size of 16 × 8.

The live mice were then anesthetized and placed in prone position, perpendicular to the axis of scanning, with the hind legs extending away from the body (Fig. 3). These scans were then analyzed to calculate the BMDs as detailed below.

### Study protocol

After 2 weeks of acclimation, basal (0 weeks) measurements were obtained from the mice as follows. The mice were weighed and anesthetized with pentobarbital (Nembutal) 50 mg/Kg. Then, whole body scans were done twice in succession with repositioning between scans. Multiple investigators positioned the animals. After each scan was obtained, the scan was evaluated for proper vertical orientation of the spine. Bone mineral densities were then calculated for these scans in the region of the Lumbar spine (the standard size of the region of interest (referred to as ‘the box’) adjusted to 16 × 8 pixels). The mice were then allowed to recover and transferred to their cages.

The mice were weighed every week. A second set of scans was obtained at 8 weeks. Again, mice were anesthetized with pentobarbital and two whole body scans were taken for each mouse, with repositioning between scans. Multiple investigators positioned the animals. After each scan was obtained, the scan was evaluated for proper vertical orientation of the spine. These scans were used to calculate the bone mineral densities for the Lumbar spine (the standard box size adjusted to 18 × 9 pixels) for the 8-week period. The mice were then allowed to recover and were transferred to their cages. The first set of animals (*n* = 8) was then sacrificed. The lumbar spine was removed by dissection and scanned by DXA. The femurs were isolated and ash weights were determined according to the procedure described by Fogelman and Wahner (1994).

### BMD computation

In order to compute the BMD of the lumbar spine, we used the follwing method of analysis. For the basals (0 weeks), a box size of 16 × 8 pixels was used, and, at the 8-week time point, a size of 18 × 9 pixels was used (accommodating for the growth of the animals). The following procedure describes the steps used to compute the BMDs.

1. Adjust the display window to provide optimal viewing of the DEXA scan for your machine.
2. Select a box size for analysis (basals = 16 × 8, 8 weeks = 18 × 9).
3. Place the box just one or two pixels above the iliac crest, which appears as a bulge in the lumbar spine. Care should be taken to place the box within the soft tissue (not including subcutaneous fat) of the mouse.
4. Manually select a region to analyze which is one pixel wide and runs through the entire length of the box, parallel to the spine, starting at the left-most border of the box.
5. Analyze the scan and record the results.
6. Repeat this analysis selecting a region to analyze which is located one pixel to the right of the previous analysis, record the results.
7. Continue to perform analyses of one pixel-wide strips until the entire box has been covered.
8. The highest BMD value obtained is recorded as the BMD for the scan.

### Data analysis

Inter-observer variability, repeat-scan variability, variability across scans of different animals, and change over time in the same animal were calculated using standard deviation and root mean square CV to determine the precision of the results. The mice from the first set were sacrificed, and BMD of the excised spine was measured. Correlations between the BMD values measured using our technique and those measured from the excised spines were calculated using linear regression analysis to assess the accuracy of our technique.

## Results

We assessed the precision of our method of BMD analysis in mice in-vivo by calculating inter-observer variability, repeat-scan variability, variability across scans of different animals, and variability over time.

### Inter-observer variability

**Figure 4:**
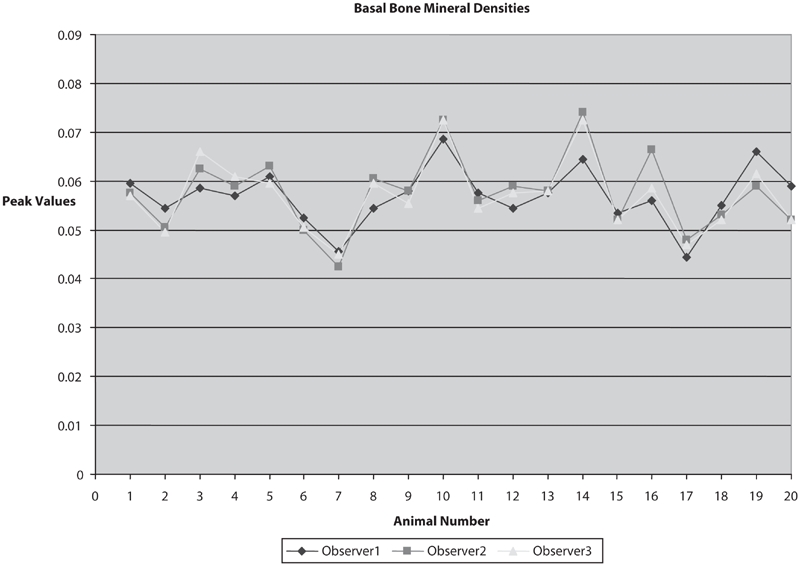
Basal BMDs. Data showing a high degree of correlation of calculated BMD between multiple observers with different experience levels (n = 20).

**Figure 5:**
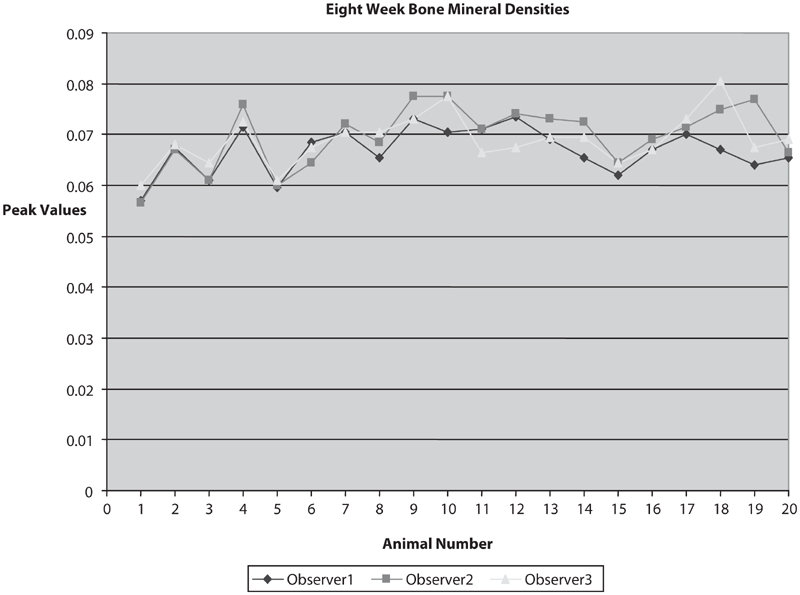
Eight week BMDs data showing a high degree of correlation of calculated BMD between multiple observers with different experience levels (n = 20).

Our data showed a high degree of correlation of calculated BMD between multiple observers with different experience levels and education (a medical doctor [observer 1], a senior college student [observer 2], and a high school student [observer 3]). Each of the 80 scans (20 mice, two time points, two scans per time point) was analyzed by three different observers. The overall mean value for the scans was 0.063 g/cm^2^. The standard deviation across observers was 0.00317 g/cm^2^, and the coefficient of variation across observers was 5.05 (%CV). All of the variability could be attributed to the manual placement of the region of interest box within the mice soft tissue for the lumbar region of the spine. Fig. 4 and Fig. 5 show the variation across observers for the same animals in the two sets, respectively.

### Repeat-scan variability

The repeat-scan variability was computed to identify the variation between the BMD of two successive scans of the same mouse. Duplicate scans were taken from 20 mice at both baseline and 8 weeks, and these scans were analyzed by three different individuals (total of 240 scan analyses). The average reading for each scan across observers was determined, and the standard deviation was then calculated for the duplicate scans. The overall mean value for repeat scans was again 0.063 g/cm^2^, the standard deviation across repeat scans was 0.00364 g/cm^2^, and the coefficient of variation across repeat scans was 5.94 (%CV). The results thus show a high degree of reliability between repeated scans, similar to the inter-observer variability.

### Variability of BMD between different animals

To assess the biological variability in BMD within a set of mice, we averaged all scan readings for each set at each time point and calculated group means and standard deviations. At week 0, the first set of mice (*n* = 8) showed an average BMD of 0.057 ± 0.006397 g/cm^2^ (mean ± SD) and 11.48 (%CV), while, at week 8, the average BMD was 0.066 ± 0.005483 g/cm^2^ (mean ± SD) and 8.33 (%CV). Similarly, for the second set of mice (*n* = 12), at week 0, the average BMD was 0.058 ± 0.007457 g/cm^2^ (mean ± SD) and 12.80 (%CV), and, at week 8, the average BMD was 0.070 ± 0.004183 g/cm^2^ (mean ± SD) and 5.94 (%CV). The average of the variation seen between different mice within the same group was 0.00588 g/cm^2^ (SD) and 9.64 (%CV). Thus, the biological variability was approximately double the variability introduced by the measurement technique.

### Variability over time

The average of all readings of the two duplicate scans for each mouse in set 1 (*n* = 8) was calculated at week 0, and a similar average was then calculated for the same mouse at week 8. The results for each mouse at week 0 were then subtracted from those from week 8. The %CV and standard deviation of the resultant data were then computed (mean ± SD = 0.012 ± 0.00798 g/cm^2^, %CV = 65.99). The same procedure was repeated for the mice in set 2 (*n* = 12), resulting in a value of 0.010 ± 0.00971 g/cm^2^, %CV = 95.38. The average standard deviation and %CV across the two sets was 0.011 ± 0.00885 g/cm^2^ (mean ± SD) and 80.68 (%CV) (Table 1). Interestingly, the biological variability in change in BMD over time exceeded not only the variability introduced by the measurement technique, but also the variability seen between different animals at a given time point.

**Table 1:** Statistical variations for region of the ‘lumbar spine of mice in vivo’ using hologic QDR-1000 plus (DXA).

	Mean ± SD (g/cm^2^)	%CV
Inter observer variation	0.063 ± 0.0317	5.05
Repeat scan variation	0.063 ± 0.00364	5.94
Change within groups	0.063 ± 0.00588	9.64
Change over-time	0.011 ± 0.00885	80.68

### Compilation of results

**Figure 6:**
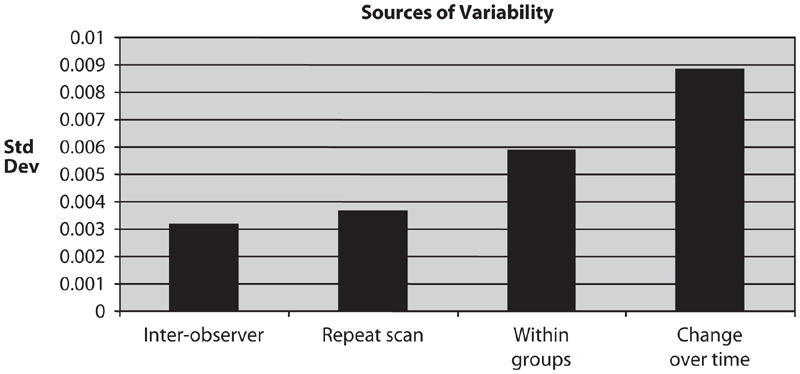
Sources of variability. The inter-observer standard deviation and the repeat-scan standard deviation shows the variation in data obtained, indicating the variation introduced by the technique of measuring BMD. The other two bars in the graph (within groups and change over time) show the biological variation in BMD in mice.

A compilation of results from this study is shown in Fig. 6, which shows the standard deviations computed in this study. The inter-observer standard deviation shows the variation introduced by data analysis, and the repeat-scan standard deviation shows the variation introduced by data acquisition. Together, these two indicate the variation introduced by the technique of measuring BMD. The other two bars in the graph (within groups and change over time) show the biological variation in BMD in mice. The standard deviations of ‘inter-observer’ and ‘repeat scans’ are much lower than those of ‘within groups’ and ‘change over time’, indicating that the error introduced by the technique is smaller than biological variability.

### Determination of accuracy

We analyzed the BMD of the excised spine of mice in set 1 (*n* = 8) at the week-8 time point and compared these measurements with the whole body scan results to determine the accuracy of our technique, as compared to this standard. As shown in Table 2, the average BMDs for the excised spine was 0.065 g/cm^2^, and the standard deviation was 0.0039. The average peak BMD for mice calculated from whole-body scans (0.066 g/cm^2^) was nearly identical to that calculated from the excised spine (0.065 g/cm^2^). The measurements obtained through our technique thus appear to be as accurate as those obtained from excised spines.

**Table 2:** Comparison of whole body scans versus excised spine.

	Mean ± SD (*n* = 8) g/cm^2^
Whole body scan	0.066 ± 0.0071
Excised spine	0.065 ± 0.0039

**Figure 7:**
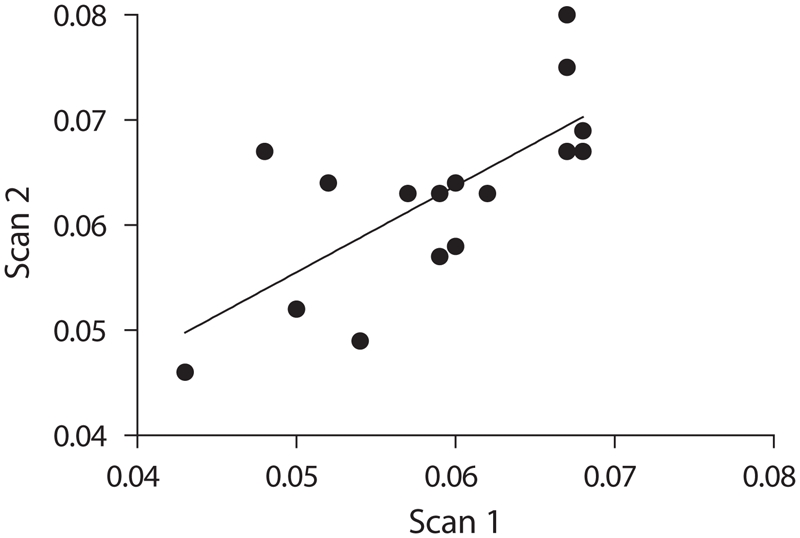
Correlation. First scan correlated with the BMD reading of the second scan (*R*^2^ = 0.5122, *p* = 0.0018).

We further analyzed the data to determine if there was any correlation between the BMD of successive scans of the same animal. As shown in Fig. 7, the BMD reading of the first scan did correlate with the BMD reading of the second scan (*R*^2^ = 0.5122, *p* = 0.0018). The ability to correlate the BMD readings of successive scans in the same animal also suggests that our method is valid.

**Figure 8:**
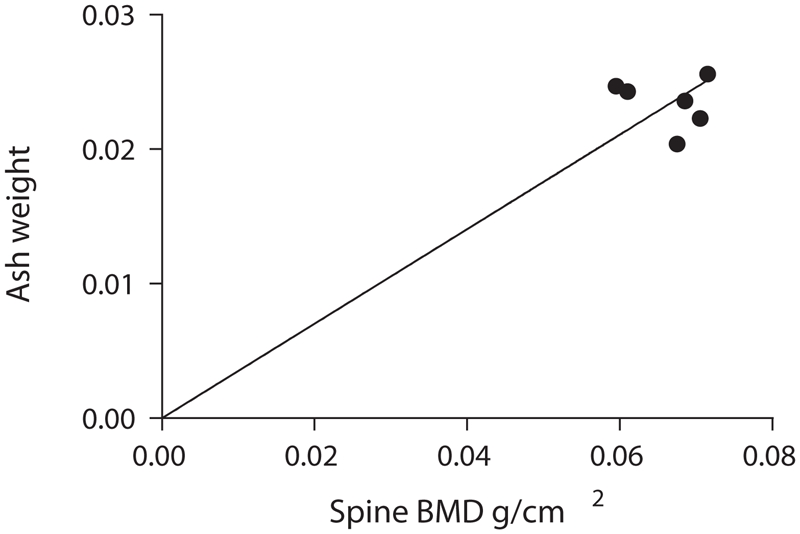
Ash weights versus whole body lumbar spine BMDs.

Lastly, the results of ash weights showed a very high significance (*p* < 0.0001) and correlated well with the whole body lumbar spine BMD values, thereby indicating that our method is accurate compared to this standard (Fig. 8).

### Assessment of repeated-measures study designs

**Figure 9:**
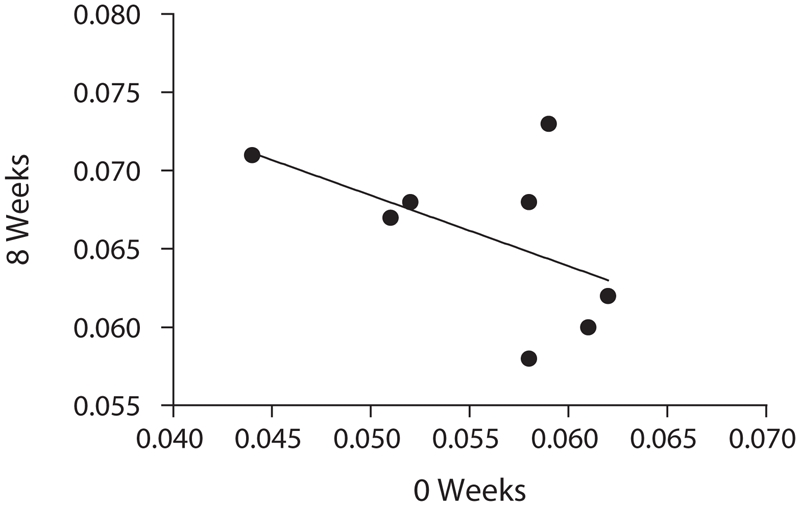
Change over time. The BMD measurement of a given mouse at week 0 was correlated with the BMD measurement of the same mouse at week 8 (*R*^2^ = 0.2691, *p* = 0.1878, NS).

We performed an analysis to determine if a repeated-measures study design would increase the power of studies to detect differences in BMD after some intervention. The average BMD measurement at week 0 and week 8 for each mouse was determined. The results were plotted against each other and the correlation coefficient was calculated. Surprisingly, we found that there was no correlation between the BMD measurement at week 0 and that of week 8 (*R*^2^ = 0.2691, *p* = 0.1878, NS) (Fig. 9), suggesting that repeated-measures study designs would not increase the power to detect changes in BMD.

## Discussion

In this paper, we propose a novel method of BMD analysis of mice in vivo for the region of the lumbar spine that has a high degree of precision and reproducibility, with good correlation between multiple observers. This analysis was done using a high resolution Hologic QDR-1000 plus machine. The results show that a regular DXA machine can be used with this technique to analyze the BMDs of the mice effectively, without using specialized software like the PIXImus scanner, which can be more expensive. The %CV for the repeated scans was 5.94% using Hologic QDR-1000 plus with our method, which is slightly better than the %CV (6.34) calculated using PIXImus (Iida-Klein et al. 2003). The inter-observer variability %CV (5.05) was higher, which could be because of the manual placement of the box within the soft tissue of the mice (ROI = lumbar spine), when compared to the PIXImus, which is already calibrated and automated.

Using this technique with a clinical DXA, it is easy for multiple individuals to analyze the scans from the same experiment. This BMD analysis was done by multiple individuals with different levels of education (a medical doctor, a senior college student, and a high school student) and without any prior experience or certification in analyzing bone mineral density. Furthermore, multiple individuals could position the animals for data acquisition, as the scans could be evaluated for proper animal placement while the animal was still anesthetized and a repeat scan could easily be obtained.

To study the effectiveness of the technique, different statistical measurements, such as inter-observer variability, repeat-scan variability, variability across scans of different animals, and variability over time were assessed. The inter-observer variability (0.063 ± 0.00317 g/cm^2^ [mean ± SD], 5.05 [%CV]) and repeat-scan variability (0.063 ± 0.00364 g/cm^2^ [mean ± SD], 5.94 [%CV]), which show the technical variations, were very low compared to the variability across scans of different animals (0.063 ± 0.00588 g/cm^2^ (mean ± SD), 9.64 [%CV]) and variability over time (0.011 ± 0.00885 g/cm^2^ (mean ± SD), 80.68 [%CV]), which show the biological variation. From Fig. 6, it can be seen that the error introduced by data acquisition and data analysis is smaller than the biological variability. The biological variations for scans across different animals and change over time cannot be minimized. Furthermore, the variability seen in a given animal over time is much greater than the variability seen between animals at a given time.

Accuracy was determined by comparing the average of the peak BMDs for the mice in-vivo (0.066 g/cm^2^) with the average BMD for the excised spines (0.065 g/cm^2^). The mean values are almost the same, thereby establishing the accuracy of the method. Further, the accuracy of our developed method is evident by the results of the bone ash weights compared with the whole body lumbar spine BMDs, which showed a very high significance (*p* < 0.0001). Results of previous investigators (Nagy et al. 2001; Iida-Klein et al. 2003) also showed that bone ash measurements can be used as a reliable measure of accuracy. Thus, BMD can be assessed in mice without the need to sacrifice the animals. We also analyzed the data to determine if there was any correlation between the BMD of successive scans of the same animal. As a further assessment of accuracy, the BMD readings of the two successive scans obtained in each animal were found to correlate well with each other (*R*^2^ = 0.5122, *p* = 0.0018) (Fig. 7). While one might have expected the BMD measurement of a given mouse at week 0 to correlate with the BMD measurement of the same mouse at week 8, we found this was not the case (*R*^2^ = 0.2691, *p* = 0.1878, NS) (Fig. 9). In fact, as shown earlier, the biological variability in BMD over time for a given mouse exceeds the biological variability between mice of the same age. It therefore appears that use of a repeated-measures study design would not increase the power of a study to detect changes in BMD.

In conclusion, we propose a new method of BMD analysis performed using a clinical DXA machine, which provides reliable and accurate results. Unlike previous techniques, this new technique is objective and is less dependent upon image quality. The data acquisition and analysis can be performed by multiple individuals with different levels of education, and, since there is no possibility of introducing bias into the BMD measurements, the analysis need not be performed in a blinded fashion. This allows the number of animals in a given study to be larger, resulting in increased power to detect differences in BMD between different sets of mice. The scans are performed on whole animals, not on excised bones. Animals can be recovered after the DEXA measurement, so time course studies can be more easily performed. The error introduced by the technique is less than that seen with the PIXImus scanner, which is designed specifically for use with small animals, and this error is smaller than the expected biological variability. The peak BMD values for the whole body scans were similar to the BMD values measured in excised spine, thereby indicating that the method is accurate. Bone ash measurements also correlated well with whole body lumbar BMD. We have used this technique in investigation of new compounds for treatment for osteoporosis, with good results (Ponnapakkam et al. 2007, 2008). Since this technique is objective and the values obtained are operator-independent, it is easy to learn and can be used with any available clinical DEXA machine. We therefore recommend that other laboratories employ this method to measure BMD in mice to provide a better standard for comparison across studies.

## References

[b1] Cheng S., Tylavsky F., Carbone L. (1997). Utility of ultrasound to assess risk of fracture. J. Am. Geriatr. Soc..

[b2] Di Leo C., Tarolo G. L., Bagni B., Bestetti A., Tagliabue L., Pietrogrande L., Pepe L. (2002). Peripheral quantitative Computed Tomography (PQCT) in the evaluation of bone geometry, biomechanics and mineral density in postmenopausal women. Radiol. Med. (Torino).

[b3] Fogelman I., Wahner H. (1994). The Evaluation of Osteoporosis: Dual Energy X-ray Absorbtiometry in Clinical Practice.

[b4] Gala Paniagua J., Diaz-Curiel M., de la Piedra Gordo C., Castilla Reparaz C., Torralbo Garcia M. (1998). Bone mass assessment in rats by dual energy X-ray absorptiometry. Br. J. Radiol..

[b5] Hofker M. (2003). Introduction: the use of transgenic mice in biomedical research. Methods Mol. Biol..

[b6] Iida-Klein A., Lu S. S., Yokoyama K., Dempster D. W., Nieves J. W., Lindsay R. (2003). Precision, accuracy, and reproducibility of dual X-ray absorptiometry measurements in mice in vivo. J. Clin. Densitom..

[b7] Lochmuller E. M., Jung V., Weusten A., Wehr U., Wolf E., Eckstein F. (2001). Precision of high-resolution dual energy X-ray absorptiometry of bone mineral status and body composition in small animal models. Eur. Cell Mater..

[b8] Moore D. M., Experimental Animal in Biomedical Research (1995). Mice. Care, Husbandry and Well-Being-An Overview by Species.

[b9] Nagy T. R., Clair A. L. (2000). Precision and accuracy of dual-energy X-ray absorptiometry for determining in vivo body composition of mice. Obes. Res..

[b10] Nagy T. R., Prince C. W., Li J. (2001). Validation of peripheral dual-energy X-ray absorptiometry for the measurement of bone mineral in intact and excised longbones of rats. J. Bone Miner. Res..

[b11] Ponnapakkam T., Katikaneni R., Ponnapakkam A., Miller E., Gensure R. (2008). Monthly administration of a novel parathyroid hormone-collagen binding domain fusion protein increases bone mineral density by more than 10 percent in normal mice.

[b12] Ponnapakkam T., Sakon J., Matsushita O., Gensure R. (2007). Weekly administration of a novel parathyroid hormone-collagen binding domain fusion protein increases bone mioneral density by more than 15 percent in normal mice.

[b13] Vonken W., Hofker M. (2005). Transgenic mice in biomedical research. Molecular Cell Biology and Molecular Medicine.

